# Factorial versus multi‐arm multi‐stage designs for clinical trials with multiple treatments

**DOI:** 10.1002/sim.7159

**Published:** 2016-11-02

**Authors:** Thomas Jaki, Despina Vasileiou

**Affiliations:** ^1^ Medical and Pharmaceutical Statistics Research Unit, Department of Mathematics and StatisticsLancaster UniversityLancasterU.K.

**Keywords:** factorial design, multi‐arm multi‐stage designs (MAMS), familywise error rate

## Abstract

When several treatments are available for evaluation in a clinical trial, different design options are available. We compare multi‐arm multi‐stage with factorial designs, and in particular, we will consider a 2 × 2 factorial design, where groups of patients will either take treatments A, B, both or neither. We investigate the performance and characteristics of both types of designs under different scenarios and compare them using both theory and simulations. For the factorial designs, we construct appropriate test statistics to test the hypothesis of no treatment effect against the control group with overall control of the type I error. We study the effect of the choice of the allocation ratios on the critical value and sample size requirements for a target power. We also study how the possibility of an interaction between the two treatments A and B affects type I and type II errors when testing for significance of each of the treatment effects. We present both simulation results and a case study on an osteoarthritis clinical trial. We discover that in an optimal factorial design in terms of minimising the associated critical value, the corresponding allocation ratios differ substantially to those of a balanced design. We also find evidence of potentially big losses in power in factorial designs for moderate deviations from the study design assumptions and little gain compared with multi‐arm multi‐stage designs when the assumptions hold. © 2016 The Authors. Statistics in Medicine Published by John Wiley & Sons Ltd.

## 1 Introduction

Despite the increased understanding of many diseases unfolding in recent years and the increased spending in research and development, the US Food and Drug Administration identified a slowdown in the approval of innovative medical therapies 1 and called for methods that achieved reliable results more quickly. It is well known that clinical trials are expensive 2, 3, and that only a small proportion (about 10*%*) of new drugs in phase I trials reaches the market 4. Moreover, phase II clinical trials designed to assess the treatment's therapeutic capacity and safety to warrant further testing in a phase III trial do not necessarily succeed in identifying potentially effective treatments, because on average only about 50*%* of the large phase III confirmatory trials are successful 5. Studying two or more treatments simultaneously within one trial is more efficient compared with the traditional separate treatment evaluation, as both the sample size and the duration of the trial will be smaller. Combination therapies are also of interest especially when monotherapies fail to prove their effectiveness or when agents are known to have different mechanisms of efficacy. Examples of such therapies include medications for the treatment of hypercholesterolemia where there is a class of cholesterol‐lowering agents that inhibit the intestinal absorption of cholesterol and another class (statins) which inhibit cholesterol biosynthesis 6.

Couper *et al.* 7 explicitly discuss the use of factorial designs in order to investigate interactions in combination treatment studies. A factorial experiment is one that two or more experimental factors are studied simultaneously 8. Specifically a 2 × 2 factorial model is one that has two factors and two levels for each factor. The core assumption for the accurate estimation of the main treatment effects is the absence of an interaction between the two treatments which directly implies that the combination of both treatments has a truly additive effect.

As an alternative to factorial designs a multi‐arm multi‐stage (MAMS) trial design (e.g. 9, 10) could be used for testing a number of new agents and their combinations at the same time. A multi‐arm (MA) design is a clinical trial design that allows the simultaneous assessment of a number of experimental treatments, which can be either different treatments or combinations of treatments, against a single control arm [11, Chapter 16]. MAMS designs are a class of the MA designs which utilise the group sequential methods 12, 13, 14 thus allowing multiple looks on the data.

The objective of this paper is to compare 2 × 2 factorial designs, where groups of patients will either take treatment A, B, both treatments or none, to MA designs where the three arms (A, B and A with B) are tested against control. The issues that need to be considered when planning a factorial trial have for example been discussed in the literature by Montgomery *et al.* 15 and those arising when applying MAMS methodology have been addressed in 16, 17. Our aim is to find the situations when the use of one design is preferable to the other in terms of the power or the sample size requirement.

## 2 Example

To illustrate the difference between a 2 × 2 factorial design and an MA design, we use a study on the clinical effectiveness of manual or exercise physiotherapy or both, in addition to usual care for patients with osteoarthritis (OA) of the hip or knee 18. Manual therapy is intended to modify the quality and range of the target joint and improve musculoskeletal function and pain. Exercise therapy is used for muscle strengthening, stretching and neuromuscular control and has been shown to be effective in the increase of physical function and pain reduction. However, there is little evidence regarding the effectiveness of manual treatment and the long‐term effectiveness of exercise therapy. The study has been designed as a 2 × 2 factorial randomised controlled trial, where 206 people are equally allocated to receive one of the following interventions: usual care, manual physiotherapy, exercise physiotherapy or manual and exercise physiotherapy, with each of the physiotherapies administered in nine treatment sessions each lasting 50 min. The primary outcome was change in the Western Ontario and McMaster osteoarthritis index (WOMAC) at 1‐year follow‐up. The primary endpoint was assumed to be normally distributed, and the trial sample size was chosen to detect a difference in WOMAC points of Δ = 28 for each main effect assuming a standard deviation, *σ* = 50. Using a two‐sided type I error of 5*%* (*α* = 0.05) – it was found that a total of 180 participants are necessary to detect a main effects difference of a comparison in the margins with 95*%* power, that is comparing the presence against the absence of manual therapy or exercise therapy, a within the table comparison of all of the active interventions versus control with 75*%* – that is when comparing manual, exercise or the combination therapy to control and an interaction between the interventions manual therapy and exercise therapy with 46*%* power. The study sample size allowed for 20*%* attrition and the study protocol planned for a total of 224 participants, but 11 months into the trial due to higher than anticipated retention rates sample size recalculation was performed allowing for 10*%* attrition, which reduced the required sample size to 200 and recruitment stopped at 206 patients. General linear regression adjusted for baseline WOMAC score, stratification variable of knee or hip OA and some pre‐specified potential confounding factors at baseline was used for the primary analysis. The results showed a significant difference in manual therapy versus no manual therapy and a non‐significant difference in exercise versus no exercise. However, a significant strong antagonistic interaction was found in this study questioning the appropriateness of a 2 × 2 factorial design.

## 3 Methods

In this section, we describe the methods used to compare factorial and MA designs in clinical trials with normally distributed endpoints in terms of sample size and power. We focus on superiority clinical trials where the question of interest lies in determining the efficacy of treatments A, B and their combination A and B together, denoted AB. Let *Y* be the variable that measures the endpoint of interest, and let *n* denote the sample size. Subscripts *A*,*B*,*A* *B* and 0 correspond to patient on treatment A, B, AB and control, respectively. Assuming that the response variables are normally distributed, we model $Y_{j}∼N(\mu_{j},\sigma^{2})$ with *j* = *A*,*B*,*A* *B*,0, where *μ*_*j*_ is the mean effect of the response to treatment or control. Correspondingly *n*_*j*_ is the number of patients allocated to a treatment or control. The one sided global null hypothesis family (*H*_0_) to be tested is

(1) $$H_{0}=\{H_{0A}:\mu_{A}⩽\mu_{0},\;H_{0B}:\mu_{B}⩽\mu_{0},\;H_{0AB}:\mu_{AB}⩽\mu_{0}\}$$

We want to control the probability of rejecting at least one true null hypothesis (the familywise error rate) at level *α* for both designs. Therefore, we are interested in finding a critical value *k* such that

(2) $$P_{H_{0}}(Z_{A}⩽k\cap Z_{B}⩽k\cap Z_{AB}⩽k)=1-\alpha .$$

We also intent to compare the number of participants that need to be included in a study so that a pre‐specified power level is achieved. The probability of correctly rejecting the global null hypothesis when the alternative is true should be large as this ensures a high powered test. Thus, the probability of type II error when the alternative hypothesis is true is defined by $\beta =P_{H_{1}}(Z_{A}⩽k\cap Z_{B}⩽k\cap Z_{AB}⩽k)$, with the design family of alternatives being *H*_1_:{*H*_1*A*_:*μ*_*A*_ > *μ*_0_,or*H*_1*B*_:*μ*_*B*_ > *μ*_0_,or*H*_1*AB*_:*μ*_*AB*_ > *μ*_0_}. This formula either helps define the relevant sample size that ensures control over the type I and type II errors with the use of numerical integration or gives the power 1 − *β* for a given sample size. Finally, this design alternative is specifying the union of the events *μ*_1*j*_ > *μ*_0_, for *j* = *A*,*B*,*A* *B* occurs, that is that any of the treatments *A*,*B*,*A* *B* have a bigger effect compared with control.

In the subsequent sections, we give the estimates of the treatment effects in a factorial design and describe our method of finding the distribution of the statistics relevant to the hypothesis testing which relate to the power of the test. Equivalently, the estimates of treatment effects in an MAMS design, as well as the statistics and distributions for the hypothesis tests and the resulting power of the tests are also provided.

### 3.1 Factorial design treatment effect estimation

By introducing indicator variables I_*A*_ and I_*B*_, specifying whether treatment A or B, respectively has been administered, we can express the response variable, *Y* as the following linear model *Y*_*i*_ = *β*_0_ + *β*_1_I_*Ai*_ + *β*_2_I_*Bi*_ + *β*_3_I_*Ai*_I_*Bi*_ + *ϵ*_*i*_, with the total number of participants being *n*_0_ + *n*_*A*_ + *n*_*B*_ + *n*_*AB*_, where and *i* = 1,2,…,*n*_0_ + *n*_*A*_ + *n*_*B*_ + *n*_*AB*_. Subscript *i* corresponds to patient *i*, and ***Y***,**I**_***A***_,I_***B***_ denote the vectors of the random variable values for all study participants. Additionally, $\epsilon_{i}∼N(0,\sigma^{2})$ and $\text{Cov}(\epsilon_{i},\epsilon_{i}^{*})=0$ for *i* ≠ *i* ^∗^.

The underlying true effect of a 2 × 2 factorial model in terms of the linear model parametrisation is illustrated by Table 1. Therefore, the hypothesis testing regarding the treatment effects *μ*_*j*_ described previously may be conducted through testing linear combinations of the coefficients ***β*** = (*β*_0_,*β*_1_,*β*_2_,*β*_3_) of the linear model.

**Table 1:** The mean response for all treatment combinations in a 2 × 2 factorial experiment.

		**B**	
**Treatments**		Presence	Absence
**A**	Presence	*β*_0_ + *β*_1_ + *β*_2_ + *β*_3_	*β*_0_ + *β*_1_
	Absence	*β*_0_ + *β*_2_	*β*_0_

Most factorial designs, despite the possibility that there may be an interaction between the two single treatments, make the assumption that there is no interaction present between the treatments, that is *β*_3_ = 0. Thus, in effect, the nested linear model in Equation ((3)) is used.

(3) $$Y_{i}=\beta_{0}+\beta_{1}I_{Ai}+\beta_{2}I_{Bi}+\epsilon_{i}$$

Denoting the design matrix of the linear model in Equation ((3)) by ***X*** = (**1**,**I_*A*_**,**I_*B*_**), then the maximum likelihood estimator for the vector of parameters can be found as $\hat{\beta }={\left(X^{⊤}X\right)}^{-1}X^{⊤}Y$; and the variance covariance matrix of the estimates is $\text{Cov}(\hat{\beta })=\sigma^{2}{\left(X^{⊤}X\right)}^{-1}$ 19 so that $\hat{\beta }∼N\left(\right.\beta ,\sigma^{2}{\left(X^{⊤}X\right)}^{-1})$. The treatment effects of interest are a linear combination of these parameters (as shown in Table 1). The distribution of a linear combination ***c*** of the coefficients ***β*** is $c^{⊤}\hat{\beta }∼N\left(\right.c^{⊤}\beta ,\sigma^{2}c^{⊤}{\left(X^{⊤}X\right)}^{-1})c)$, with (***X*** ^⊤^ ***X***)^ − 1^ given by Equation ((A1)) in the Appendix.

Assuming that *β*_3_ = 0 then yields that the treatment effects for A, B and AB are *β*_1_,*β*_2_ and *β*_1_ + *β*_2_, respectively and correspondingly $c_{A}^{⊤}=(0,1,0),c_{B}^{⊤}=(0,0,1)$ and $c_{AB}^{⊤}=(0,1,1)$.

#### 3.1.1 Joint comparison for both sole treatments and their combination to placebo

The relevant statistics for the hypothesis tests in Equation ((1)) are based on the formula of Equation ((4)).

(4) $$Z_{c_{j}^{⊤}\beta }=\frac{c_{j}^{⊤}\hat{\beta }}{\sigma \sqrt{c_{j}^{⊤}{\left(X^{⊤}X\right)}^{-1}c_{j}}}$$

The statistic to test for an effect of treatment *A* for *j* = *A*,*j* ^∗^ = *B* and vice versa is given by $Z_{A}=\frac{\frac{n_{0}n_{A}}{n_{0}+n_{A}}({\bar{Y}}_{A}-{\bar{Y}}_{0})+\frac{n_{AB}n_{B}}{n_{B}+n_{AB}}({\bar{Y}}_{AB}-{\bar{Y}}_{B})}{\sigma \sqrt{\frac{n_{0}n_{A}}{n_{0}+n_{A}}+\frac{n_{B}n_{AB}}{n_{B}+n_{AB}}}}$ and $Z_{B}=\frac{\frac{n_{0}n_{B}}{n_{0}+n_{B}}({\bar{Y}}_{B}-{\bar{Y}}_{0})+\frac{n_{AB}n_{A}}{n_{A}+n_{AB}}({\bar{Y}}_{AB}-{\bar{Y}}_{A})}{\sigma \sqrt{\frac{n_{0}n_{B}}{n_{0}+n_{B}}+\frac{n_{A}n_{AB}}{n_{A}+n_{AB}}}}$ with ${\bar{Y}}_{j},\;j=A,B,AB\;\text{or}\;0$  denoting the sample means of all observations in the *j*th treatment group. These are the statistics typically used in factorial experiments (e.g. 8). Notice that the aforementioned statistics include information from the sole treatment arms and also from the combination arm as, under the assumption of additivity, one can extract the information about each individual arm.

To test the effect of the combination treatment, the standard test statistic $Z_{AB}=\frac{{\bar{Y}}_{AB}-{\bar{Y}}_{0}}{\sigma \sqrt{\frac{n_{0}+n_{AB}}{n_{0}n_{AB}}}}$ is used, instead of that derived using Equation ((4)) and ***c***_*AB*_ which relies heavily on the additivity of the sole treatment effects and the absence of any interaction between them. Information about the combination treatment in the full model comes only from patients on that arm.

To simplify notation and recognising that at the planning stage unequal numbers in the single treatment group are unlikely, we assume that the allocation ratios for the single treatment groups relative to the control group is *r* and the allocation ratio in the combination treatment group relative to control is *q*. Under these assumptions, *n*_*A*_ = *n*_*B*_ = *r* *n*_0_ and *n*_*AB*_ = *q* *n*_0_, which result to the simplified z‐statistics shown by Equation ((5)).

(5) $$\begin{array}{cc} Z_{A} & =\sqrt{n_{0}}\frac{r(r+q)({\bar{Y}}_{A}-{\bar{Y}}_{0})+qr(1+r)({\bar{Y}}_{AB}-{\bar{Y}}_{B})}{\sigma \sqrt{\left(1+r)(r+q)(r^{2}+2rq+r^{2}q\right)}}\\ Z_{B} & =\sqrt{n_{0}}\frac{r(r+q)({\bar{Y}}_{B}-{\bar{Y}}_{0})+qr(1+r)({\bar{Y}}_{AB}-{\bar{Y}}_{A})}{\sigma \sqrt{\left(1+r)(r+q)(r^{2}+2rq+r^{2}q\right)}}\\ Z_{AB} & =\sqrt{n_{0}}\frac{{\bar{Y}}_{AB}-{\bar{Y}}_{0}}{\sigma \sqrt{\frac{1+q}{q}}} \end{array}$$

#### 3.1.2 Designing a factorial study with familywise error rate control

In a 2 × 2 factorial design, usually three different hypothesis are tested, and hence, it is of interest to avoid an inflation of the overall type‐I error. More specifically, we wish to ensure that the familywise error rate (FWER) defined as *P*(reject at least one *H*_0*j*_ incorrectly) is controlled at a pre‐specified level *α*. To determine the FWER, the joint distribution of the test statistics is necessary. Because the test statistics are marginally normal, it can be shown that they jointly follow a trivariate normal distribution (Appendix B.1). We can use this result to find the parameters of this normal distribution under the null hypothesis and determine an overall critical value for all comparisons that controls the FWER at a pre‐specified level alpha.

In particular, because $\text{Cov}({\bar{Y}}_{A},{\bar{Y}}_{B})=0,\text{Cov}({\bar{Y}}_{A},{\bar{Y}}_{AB})=0$ and $\text{Cov}({\bar{Y}}_{B},{\bar{Y}}_{AB})=0$, then Var(*Z*_*A*_) = Var(*Z*_*B*_) = Var(*Z*_*AB*_) = 1. Further, because of the symmetry of the statistics *Z*_*A*_,*Z*_*B*_, it is easily shown that Cov(*Z*_*A*_,*Z*_*AB*_) = Cov(*Z*_*B*_,*Z*_*AB*_). Under the null hypothesis, the joint distribution of the statistics is a trivariate normal density with mean zero and variance covariance matrix *V*, which is shown in Equation ((6)), and the special case of a balanced design (*r* = *q* = 1) is given in Appendix B.1, Equation ((B4)).

(6) $$V=\left(\begin{array}{lll} 1 & \frac{r{\left(r+q\right)}^{2}+qr{\left(1+r\right)}^{2}-2q(1+r)(r+q)}{\left(1+r)(r+q)(r+2q+rq\right)} & \frac{\sqrt{rq}(1+2r+q)}{\sqrt{\left(1+r)(1+q)(r+q)(r+2q+rq\right)}}\\ \frac{r{\left(r+q\right)}^{2}+qr{\left(1+r\right)}^{2}-2q(1+r)(r+q)}{\left(1+r)(r+q)(r+2q+rq\right)} & 1 & \frac{\sqrt{rq}(1+2r+q)}{\sqrt{\left(1+r)(1+q)(r+q)(r+2q+rq\right)}}\\ \frac{\sqrt{rq}(1+2r+q)}{\sqrt{\left(1+r)(1+q)(r+q)(r+2q+rq\right)}} & \frac{\sqrt{rq}(1+2r+q)}{\sqrt{\left(1+r)(1+q)(r+q)(r+2q+rq\right)}} & 1 \end{array}\right)$$

To determine the critical value *k* to ensure FWER control, one can therefore numerically search for the value of *k* that satisfies Equation ((2)) using the distributional results given here.

Under the alternative hypothesis, the joint distribution of the statistics changes, and the trivariate normal is no longer centred at 0; however, the variance‐covariance matrix of the distribution remains the same. The mean of the distribution under the alternative is given by Equation ((7)).

(7) $$E\left(\begin{array}{l} Z_{A}\\ Z_{B}\\ Z_{AB} \end{array}\right)=\left(\begin{array}{l} \frac{\sqrt{rn_{0}}\left(\right.(r+q)(\mu_{A}-\mu_{0})+q(1+r)(\mu_{AB}-\mu_{B}))}{\sigma \sqrt{\left(1+r)(r+q)(r+2q+qr\right)}}\\ \frac{\sqrt{rn_{0}}\left(\right.(r+q)(\mu_{B}-\mu_{0})+q(1+r)(\mu_{AB}-\mu_{A}))}{\sigma \sqrt{\left(1+r)(r+q)(r+2q+qr\right)}}\\ \frac{\sqrt{qn_{0}}(\mu_{AB}-\mu_{0})}{\sigma \sqrt{1+q}} \end{array}\right)$$

### 3.2 Multi‐arm multi‐stage designs

Multi‐arm multi‐stage designs evaluate the effect of several treatments in one trial by testing more than one hypothesis simultaneously for times up to the maximum number of stages. The special case of such a trial with three active treatments and the additional restriction of the third arm being assigned to the combination treatment is the closest equivalent to a 2 × 2 factorial design, where the null hypothesis is in the form of Equation ((1)). In essence MA trials treat each arm independently when estimating treatment effects rather than learning something about the individual treatments through the combination arm as in a factorial design. It still remains possible to make observations on the relationship between the individual treatments by reviewing their joint effect on the combination arm. Further to that, to enable a comparison between them, it is necessary to control the FWER at the same level, *α*, which is used for the factorial design hypothesis testing.

#### 3.2.1 Single stage multi‐arm design

Effectively the test statistics in an MA design with one stage can be viewed as the result of using formula ((4)) for the complete model which includes an interaction term. Specifically, the statistic for the comparison of the *j*th active treatment to control in one‐stage design is defined as in Equation ((8)).

(8) $$Z_{j}^{{}^{\prime }}=\frac{{\bar{Y}}_{j}-{\bar{Y}}_{0}}{\sigma \sqrt{\frac{n_{0}+n_{j}}{n_{0}n_{j}}}},\;\text{with}\;j=A,B,AB.$$

The joint distribution of the statistics under the null hypothesis is a trivariate normal density with 0 mean and variance covariance matrix $W=\left(\begin{array}{lll} 1 & \frac{r}{1+r} & \frac{\sqrt{rq}}{\sqrt{\left(1+r)(1+q\right)}}\\ \frac{r}{1+r} & 1 & \frac{\sqrt{rq}}{\sqrt{\left(1+r)(1+q\right)}}\\ \frac{\sqrt{rq}}{\sqrt{\left(1+r)(1+q\right)}} & \frac{\sqrt{rq}}{\sqrt{\left(1+r)(1+q\right)}} & 1 \end{array}\right)$, where *n*_*A*_ = *n*_*B*_ = *r* *n*_0_ and *n*_*AB*_ = *q* *n*_0_.

Using this distribution, the critical value *k* can be obtained in the same manner as for the factorial design. Note that this test is a Dunnett test 20.

#### 3.2.2 Two‐stage multi‐arm design

Finally, we also compare the factorial design with an MA design with two stages, where we assume that *r* = *q*, that is the allocation ratio is the same between all active treatments and control. Extensions to more stages and other allocation ratios are possible 21, 22, but for simplicity, we focus on two‐stage designs only. A two‐stage design is a sequential design where by one is allowed to examine the data at a specific time point or after a defined number of patients have been followed up, based on a stopping rule derived from repeated significance tests. Group sequential designs allow for early stopping of the trial, either because of efficacy or futility, whilst still fully controlling the pre‐specified type I error 11. At the interim analysis, the test statistics are compared against pre‐determined boundaries. If at least one test statistic exceeds the upper boundary (*u*), the null hypothesis can be rejected and the study stopped. If the study can not be stopped for efficacy, any treatment whose test statistic falls below the lower bound (*l*) will be dropped from the remainder of the study. If all treatments are dropped, the study is stopped. Note that, although a similar strategy could be conceived for a factorial design, we do not consider such a design as dropping an arm in a factorial design impacts on the arms one is still interested in.

In this multi‐stage design, the set of previously stated null hypotheses in Equation ((1)) is potentially tested twice. Because of making multiple comparisons, we need to control the familywise error rate, *α*, and we use the multiple testing procedure for multiple stages described by 21 which is the multi‐stage extension of the Dunnett test used for the single stage design 20. In this type of multi‐stage clinical trial designs fixing the type I error and power, is not sufficient for their full specification. The probability of rejecting the null hypothesis depends on the stopping boundaries at each stage which need to be specified. Lower boundaries are used to stop a treatment whose test statistic falls below the threshold, and upper boundaries are used to stop the trial when any test statistic exceeds this boundary, as a treatment that is superior to control is found. In our two‐stage design, we use the O'Brien–Fleming boundary shape 23 for *u*_1_,*u*_2_ and a fixed lower boundary at 0, that is *l*_1_ = 0. The actual type I error equation in this two‐stage design is specified as

$$\begin{array}{cc} \alpha & =1-\overset{\infty }{\underset{-\infty }{\int }}\overset{\infty }{\underset{-\infty }{\int }}\left[\Phi \left(\right.t_{2})+\Phi_{2}\left(u_{1}\sqrt{2}+t_{2},u_{2}\sqrt{2}+\frac{t_{1}+t_{2}}{\sqrt{2}},{\left(\sqrt{2}\right)}^{-1}\right)\right.\\ \;\;\;\;\;{\left.-\;\Phi_{2}\left(t_{2},u_{2}\sqrt{2}+\frac{t_{1}+t_{2}}{\sqrt{2}},{\left(\sqrt{2}\right)}^{-1}\right)\right]}^{3}d\Phi (t_{1})d\Phi (t_{2}) \end{array}$$

where Φ denotes the standard normal distribution function and $\Phi_{2}(a,b,{\left(\sqrt{2}\right)}^{-1})$ denotes the result of the integration of a bivariate standard normal density with covariance ${\left(\sqrt{2}\right)}^{-1}$ over region [*a*,*b*]. To ensure control of the type II error, we need to be able to reject the null hypothesis if the mean treatment response is large, and for that purpose, we employ the least favourable configuration (LFC) to specify the power of the design, which is defined as the following probability *P*(*R* *e* *j* *e* *c* *t* *H*_0*AB*_|*μ*_*AB*_ = Δ,*μ*_*A*_ = *δ*_0_,*μ*_*B*_ = *δ*_0_). The specific equation for our implemented two‐stage design given the effect sizes Δ,*δ*_0_ once more involves two‐dimensional integration and is shown here.

$$\begin{array}{cc} 1-\beta & =\overset{\infty }{\underset{-\infty }{\int }}\Phi \left(u_{1}\sqrt{2}+t+\frac{\sqrt{n_{0}}}{\sigma }\Delta \right){\left[\Phi \left(t+\frac{\sqrt{n_{0}}}{\sigma }(\Delta -\delta_{0})\right)\right]}^{2}d\Phi (t)\\ \;+\overset{\infty }{\underset{-\infty }{\int }}\overset{\infty }{\underset{-\infty }{\int }}\left[\Phi \left(\right.t_{2}-\frac{n_{0}}{\sigma }\delta_{0})\;+\Phi_{2}\left(u_{1}\sqrt{2}+t_{2}-\frac{n_{0}}{\sigma }\delta_{0},t_{1}+(\Delta -\delta_{0})\frac{\sqrt{2n_{0}}}{\sigma },{\left(\sqrt{2}\right)}^{-1}\right)\right.\\ \;{\left.-\Phi_{2}\left(t_{2}-\frac{\sqrt{n_{0}}}{\sigma }\delta_{0},t_{1}+(\Delta -\delta_{0})\frac{\sqrt{2n_{0}}}{\sigma },{\left(\sqrt{2}\right)}^{-1}\right)\right]}^{2}\\ \;\left[\;\Phi \left(\;2u_{1}+t_{2}\sqrt{2}\;-\;t_{1}\;-\frac{\sqrt{2n_{0}}}{\sigma }\Delta \;\right)\;-\Phi \left(\;t_{2}\sqrt{2}-t_{1}\;-\;\frac{\sqrt{2n_{0}}}{\sigma }\Delta \;\right)\;\right]\;\Phi \left(\right.t_{1}\;\sqrt{2}-t_{2}\;-\;2u_{2})d\Phi (t_{1})d\Phi (t_{2}) \end{array}$$

The aformentioned equations are provided here for reproducibility of our results. Detailed derivations of these equations for a general number of treatments and stages can be found in 21. We have used the R package MAMS 24 to obtain the full specification of the design.

## 4 Results

In this section, we firstly explore some of the design features of factorial designs and subsequently compare these designs to MA and MAMS designs. We begin by looking into how different allocation ratios affect properties of the factorial design. Similar evaluations of MAMS designs can be found in 17. All results in this section are based on the analytic formulae in the previous section and have been verified with 10 000 fold simulations.

A study of covariances between the test statistics of Equations ((5)) presented in matrix ((6)) demonstrates that the correlation between *Z*_*A*_,*Z*_*AB*_ is always positive and that *Z*_*A*_,*Z*_*B*_ are uncorrelated when *q* = *r* ^2^. We can show that by studying the correlation as a function of the allocation ratio *r*, specifically when *q* = *r* ^2^, we have $\text{Cov}(Z_{A},Z_{AB})=f(r)=\sqrt{\frac{r}{1+r^{2}}}$, with *r* > 0. The first derivative of this is $\frac{df(r)}{dr}=\frac{1-r^{2}}{2\sqrt{r}{\left(1+r^{2}\right)}^{3/2}}$ and is used to find the extremes of *f*(*r*). Setting the first derivative to 0, we find that *r* = 1 which maximises the function *f*(*r*) and $f(1)=1/\sqrt{2}$. Therefore, the information obtained about the treatment effects by statistics *Z*_*A*_,*Z*_*B*_ is maximised, and the additional information from statistic *Z*_*AB*_ is minimised as they are correlated to the highest level, making this setup very appealing when the design assumptions are met.

### 4.1 The different treatment allocations combination effect on the critical value

**Figure 1:**
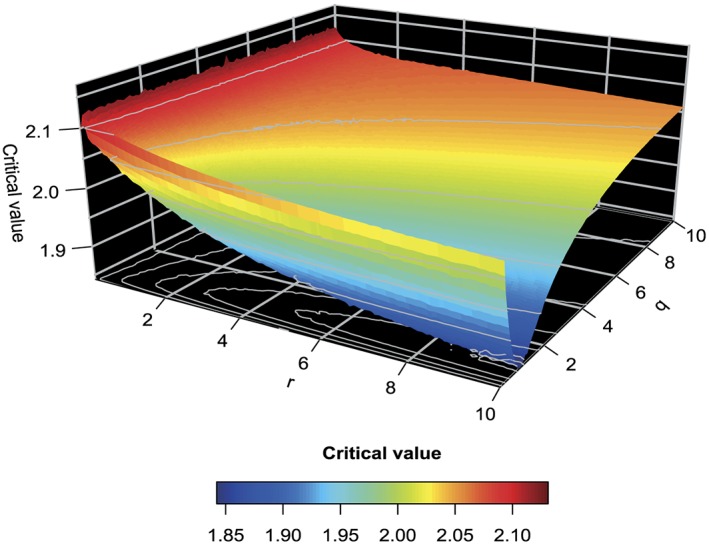
The critical values of a factorial design with varying allocation ratio when α = 0.05. A r,q grid size of 0.01/0.1, respectively is used.

The joint distribution of the test statistics in Equations ((5)) under the null hypothesis can be used to find the critical value that corresponds to the probability of committing a type I error, which we set to 5*%* here. In the case of a balanced design, the critical value is found to be *k* = 2.028. We investigated further how differences in the allocation ratios *r*,*q* affect the choice of the critical value. Figure 1 and Table 2 show the critical values for the different allocation ratio combinations of the sole experimental treatments and combination treatment. We find that the critical value varies substantially for the different allocation ratio combinations and reveals the extent to which changes in the patient recruitment ratios for each of the treatments will make it easier or more difficult to reject the global null hypothesis compared with the standard set by the balanced design.

**Table 2:** Critical value demonstration for specific allocation combination ratios r,q.

			**q**			
**r**		**0.1**	**0.5**	**1**	**1.5**	**2**
**0.1**		2.09	2.10	2.11	2.11	2.11
**0.5**		2.07	2.05	2.07	2.08	2.08
**1**		2.06	2.02	2.03	2.04	2.05
**2**		2.04	1.97	1.98	2.00	2.02

Whilst retaining the type I error rate fixed, we can use the information in Figure 1 to find the optimal choice of *q* for a given *r* in terms of minimising the critical value, which by implication ensures that the required sample size will be small. It is worth noting that whilst *r* ranges, the optimal value for *q* is largely unaffected. It seems that for any value of the allocation ratio *r* to the single treatments, the optimal choice for the allocation ratio of the combination treatment is less than 1. More specifically for the most interesting range of *r*∈[0.5,2.5], the optimal choice for *q* is 0.8, when *r* = 2.5 yielding the optimum critical value of 1.954. In other words, two and a half times as many patients should be allocated to treatments A and B compared with the control arm, whilst only 80*%* of the number on control should be devoted to the combination arm. When (numerically) searching the optimal choice of an allocation ratio when *r* = *q*, we find *r* = *q* = 1.7 to be optimal which corresponds to critical value 2.017. Note that this optimal allocation ratio is in the opposite direction with the one determined for the Dunnett test for which $\sqrt{K}$, with *K* being the number of active treatments, has been shown to be optimal 25.

### 4.2 Sample size when varying allocation ratio combinations

We now focus on the effect of varying allocation ratio combinations on the sample sizes when the alternative hypothesis scenarios are in the first instance consistent with the additivity assumptions of the factorial design and in the second when they are inconsistent with it, with the sole treatments having either a synergistic or an antagonistic effect. Throughout, we wish to control the FWER at 5*%* and seek a power of 90*%* to detect at least one superior treatment.

#### 4.2.1 Allocation ratio effect in alternative scenarios consistent with the factorial design assumptions

**Figure 2:**
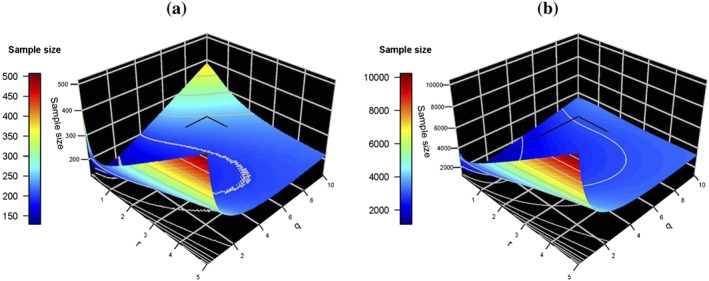
Plots (a) and (b) show the optimal total sample size that ensures power at least 0.9 for each combination of r,q when the alternative hypothesis scenarios are consistent with the factorial design assumptions. Specifically, in plot (a), any of the sole experimental treatment groups may have an interesting effect, with either μ_A_ − μ_0_ = Δ and μ_B_ − μ_0_ = δ_0_ or correspondingly μ_A_ − μ_0_ = δ_0_ and μ_B_ − μ_0_ = Δ whilst finally μ_AB_ − μ_0_ = Δ + δ_0_, and in (b) both of the sole experimental treatment groups have an uninteresting effect, with μ_A_ − μ_0_ = μ_B_ − μ_0_ = δ_0_ whilst μ_AB_ − μ_0_ = 2δ_0_.

We consider finding the optimal sample size for any design configuration in a specific range of possible allocation rations *r*,*q*, and we denote an interesting effect by Δ whilst a positive yet uninteresting effect is *δ*_0_. We deliberately consider a wide range of allocation ratios to ensure that the optimal allocation is contained in the display and also show that extreme allocation ratios do result in very large sample sizes. Figure 2 and Table 3 show the total sample size for different allocation ratios when one of the sole treatment arms has the interesting effect whilst the other sole treatment has an uninteresting effect (*μ*_*A*_ − *μ*_0_ = Δ,*μ*_*B*_ − *μ*_0_ = *δ*_0_,*μ*_*AB*_ − *μ*_0_ = Δ + *δ*_0_,*μ*_0_ = 0) in panel (a) whilst in panel (b) both sole treatments have an uninteresting effect (*μ*_*A*_ − *μ*_0_ = *μ*_*B*_ − *μ*_0_ = *δ*_0_,*μ*_*AB*_ − *μ*_0_ = 2*δ*_0_). Under the first configuration, the optimal total sample size when we assume that Δ = 0.5 and *δ*_0_ = 0.1 in the case of a balanced design is that 160 patients need to be included in the study to achieve power of at least 0.9, that is $P((Z_{A},Z_{B},Z_{AB})⩾(k,k,k)|(Z_{A},Z_{B},Z_{AB})∼MVN((\Delta ,\delta_{0},\Delta +\delta_{0}),V)⩾0.9$ . The smallest total sample size of 129 patients is achieved when *r* = 0.01 and *q* = 0.9. Clearly, such a small allocation ratio would not be useful in practice, but it does show that, under the assumptions of a factorial design, one might as well allocate no patients to the single arms as their treatment effect can be estimated from the combination arm anyway. We see that in this scenario changes in the allocation ratios do not have a big effect on the required sample size to achieve a target power. We only see notable increases in the sample size when either both *q* and *r* are very small or *q* is small whilst *r* is big and vice versa. Additionally, if we look at the sample size requirements when *δ*_0_ decreases, or is set to 0, we notice that the sample size requirements are a bit higher. For example, the number of patients to be recruited for the control group in the case of a balanced design is *n*_0_ = 43 which means that a total sample size of 172 patients need to be included in the study to achieve power of at least 0.9. In general, the range where the required sample size is big when both *q*,*r* and *q* alone are small whilst *r* increased is a bit broader.

**Table 3:** Total sample size for specific allocation combination ratios r,q as per Figure 2(a).

		**q**			
**r**	**0.1**	**0.5**	**1**	**1.5**	**2**
**0.01**	380	145	130	135	145
**0.1**	308	149	132	139	148
**0.5**	219	160	144	147	156
**1**	233	175	160	157	160
**2**	294	218	190	179	175

Looking into the alternative hypothesis when both single experimental treatments have a weak effect and the combination treatment has the addition of both of those effects (Figure 2(b) and Table 4), we find that the optimal sample size when we assumed that *δ*_0_ = 0.1 in the case of a balanced design is *n*_0_ = 502 which means that a total sample size of 2008 patients need to be included in the study to achieve power of at least 0.9. We also find the configurations which result to the smallest sample size. The minimum occurs when *r* = 0.01 and *q* = 1, which gives a total sample size of 1150 patients to be included in the study, which is about half of that required by a balanced design. In fact, the smallest sample size occurs when *r* is at the border of the grid we are considering. In general, we notice that the smallest sample sizes occur when *r*,*q* are moderately small and that the differences between the allocation combinations in terms of sample size are substantial.

**Table 4:** Total sample size for specific allocation combination ratios r,q as per Figure 2(b).

		**q**			
**r**	**0.1**	**0.5**	**1**	**1.5**	**2**
**0.01**	3455	1295	1150	1198	1291
**0.1**	3492	1404	1224	1254	1341
**0.5**	3297	1894	1572	1544	1589
**1**	3866	2475	2008	1898	1894
**2**	5404	3603	2872	2591	2475

#### 4.2.2 Allocation ratio effect when alternative hypotheses are inconsistent with the factorial design assumptions

**Figure 3:**
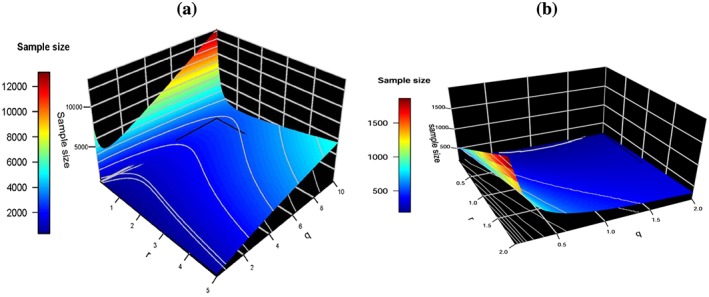
Plots (a) and (b) show the optimal total sample size that ensures power at least 0.9 for each combination of r,q in consistent with the alternative hypothesis scenarios where in (a) any sole treatment group may have an interesting effect, either μ_A_ = Δ or μ_B_ = Δ and (b) the combination treatment μ_AB_ = Δ has the interesting effect, in a least favourable configuration set up where the remaining treatment groups have an uninteresting effect δ_0_ and for the controls μ_0_ = 0.

The minimum total trial sample size requirement for the most interesting range of different combinations of allocation ratios *r*,*q*, when any of the three treatments have an interesting effect ensuring that $P(\text{Reject}\;H_{0}|H_{1j}\;\text{is true})⩾1-\beta$ and $H_{1j}^{\left(LFC\right)}:\mu_{j}=\Delta \;\mu_{j^{*}}=\delta_{0}\;\mu_{0}=0$ for *j*,*j* ^∗^∈(*A*,*B*,*A* *B*), an LFC scenario is shown in Figure 3. Specifically, Figure 3 presents two scenarios for the different combinations of allocation ratios *r*,*q* and the optimal choice of sample size necessary to detect an interesting effect Δ = 0.5, whilst assuming an uninteresting effect *δ*_0_ = 0.1 and standard deviation *σ* = 1, for a pre‐specified target power 1 − *β* = 0.9 when the assumption of additivity of the sole treatments effects to produce the combination treatment effect is not satisfied. The two alternative hypothesis scenarios are (a) H_1*A*_:*μ*_*A*_ − *μ*_0_ = Δ&*μ*_*B*_ − *μ*_0_ = *μ*_*AB*_ − *μ*_0_ = *δ*_0_ when the sole treatments interact antagonistically and (b)  *H*_1*AB*_:*μ*_*AB*_ − *μ*_0_ = Δ,*μ*_*A*_ − *μ*_0_ = *μ*_*B*_ − *μ*_0_ = *δ*_0_, when the sole treatments interact synergistically.

In Figure 3(a) and Table 5, we notice that the smallest trial sizes with 90*%* power occur when *r* is around one and *q* is small. The optimal sample size in the case of a balanced design is when *n*_0_ = 176 which means that a total sample size of 704 patients need to be included in the study to achieve at least 90*%* power. We also find what is the configuration which results in the smallest sample size. The minimum occurs when *r* = 0.81 and *q* = 0.1, which gives a total sample size of 326 patients to be included in the study which strikingly is less than half of the patients that need to be included when the design is balanced. Once again, a very extreme allocation ratio of *q* = 0.1 is found to be best. Because the additivity is violated, information on the combination arm can no longer readily be used to extract information on the single arms, and hence, fewer patients are allocated to this arm. We have found that the highest sample sizes correspond to the cases when the allocation ratios *r*,*q* result to a high negative correlation between the sole treatment statistics and a low correlation between the one of the sole treatment statistics and the combination treatment statistic. Conversely, the smallest sample sizes occur when the correlation between the z‐statistics is high. Finally, it seems that even when the assumptions of the factorial design are not satisfied, and it becomes necessary to increase the sample size compared with when they are satisfied, the allocation ratio choices have an important bearing to the choice of sample size through their control of the correlation between the statistics.

**Table 5:** Total sample size for specific allocation combination ratios r,q as per Figure 3(a).

		**q**			
**r**	**0.1**	**0.5**	**1**	**1.5**	**2**
**0.1**	1000	1547	1944	2299	2650
**0.5**	353	585	804	1000	1180
**1**	329	508	704	877	1035
**2**	388	561	766	962	1145

In Figure 3(b) and Table 6, we find that in the case of a balanced factorial design, the required sample size for the control group which achieves this power is *n*_0_ = 81, with a total sample size of 324, where as under the previously discussed alternative where only the sole experimental treatment had an interesting effect, the required sample size was *n*_0_ = 176. The configuration that gives, in this setting, the total minimum sample size of 199 occurs when *r* = 0.1 and *q* = 1. We also notice when studying the effect of the different allocation ratios on the sample size that only when *q* is very small, the sample size requirement is big and indeed bigger than that of the aforementioned LFC scenario. This becomes more prominent as *r* increases. In all other allocation ratio combinations, the sample size requirement to achieve the target power is small and in fact quite a lot smaller when compared with the requirement discussed in the aforementioned paragraph. Lastly, we note that in a factorial design where the main focus is on the sole treatments and testing if those have a significant effect, such an alternative hypothesis scenario would result to a high probability of rejecting the null hypothesis for both sole treatments and simulation study results have shown that is the case for the vast majority of allocation combinations *r*,*q*, apart from the cases when *r* and *q* are very small.

**Table 6:** Total sample size for specific allocation combination ratios r,q as per Figure 3(b).

		**q**			
**r**	**0.1**	**0.5**	**1**	**1.5**	**2**
**0.1**	606	228	196	203	215
**0.5**	890	315	252	245	253
**1**	1256	431	324	297	290
**2**	1993	664	462	387	347

In conclusion, we found that for the majority of allocation ratio combinations, the power under the first type of hypothesis when the single experimental treatments have the interesting effect is less than under the second hypothesis where the combination treatment has the interesting effect. There are some allocation ratio combinations when *q* is small that give equal power under both types of alternative hypotheses scenarios. A setup where one experimental treatment has a strong effect whilst the combination of that treatment with another having no effect implies an antagonistic effect between the treatments so that the combination has a dissimilar effect to that of the working single treatment. It therefore makes it very difficult for the other experimental treatment to demonstrate its true effect, because of the way the effect is evaluated. Additionally, we note that in the case of the combination treatment having a strong effect in the LFC that amplifies the effect of the single experimental treatments further compared with the case that *δ*_0_ = 0 thus necessitating the inclusion of more people in the study when that is the case.

### 4.3 Effect of interaction

One of the fundamental assumptions used in factorial designs to find the critical value and required sample size is the additivity of the treatment effects. To explore the impact of deviating from this assumption, we investigate a scenario where the data for each of the single experimental treatments is consistent with the null hypothesis, but the data for the combination of the treatments are drawn from a distribution which includes varying degrees of interaction between the treatments implying either an antagonist or synergistic effect or no relation between them. The strength of the interaction, *β*_3_, ranges from  − 1 (strong antagonistic effect) to 1 (strong synergistic effect).

**Figure 4:**
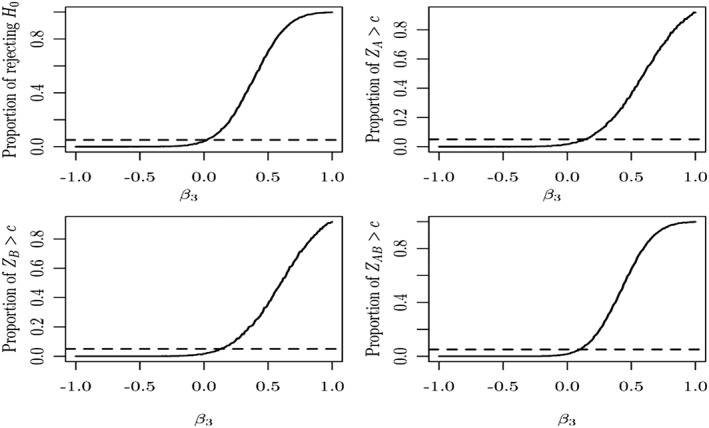
The Monte Carlo estimate of the probability of rejecting the null hypothesis based on 10 000 simulated samples when the sole experimental treatments are assumed not to have an effect (solid line) and the type I error reference line at 0.05 (dashed line). The top left graph shows the probability to reject the global null hypothesis whilst the remaining graphs provide the probabilities for the individual null hypotheses.

Figure 4 shows the empirical probability of rejecting the four null hypotheses (global null hypothesis and the three single treatment to control comparison) based on 10 000 simulations for a balanced factorial design with *β*_0_ = *β*_1_ = *β*_2_ = 0 and different levels of interaction *β*_3_. An overall type I error of *α* = 0.05 is used and *n*_0_ = *n*_*A*_ = *n*_*B*_ = *n*_*AB*_ = 50 is chosen to give power of 1 − *β* = 0.9 for Δ = 0.5,*δ*_0_ = 0.1 and standard deviation *σ* = 1. The four plots relate to the global null hypothesis given in Equation ((1)) (top left) as well as the three individual null hypotheses comparing one arm against control. We can clearly observe a notable inflation of the type I error of the single treatment arm comparison in the cases when the treatment interact in a synergistic manner (*β*_3_ > 0). It is also worth pointing out that despite the observations on the single arms being consistent with the null hypothesis, the chance to reject the hypothesis related to the single treatment arms is also increasing. This is because the observations on the combination arm are also contributing to the test statistics for the single treatment arms.

We, moreover, investigated the effect on the power of the hypothesis testing when the experimental treatments have an effect when individually administered but interact antagonistically when combined in a balanced design. We assumed that treatment A has a strong effect Δ = 0.5 and treatment B has a weaker one *δ*_0_ = 0.25, and by using a similar simulation set‐up where the rest of the design parameters are common, we find a similar pattern to Figure 4 with the curves shifted further to the left regarding the rejection probability. We found a loss of power when the treatments have in combination an antagonistic effect and a level of power close to the nominal value otherwise. Further results on the effect of the power of hypothesis testing for a broader variety of alternative scenarios are presented forwards in Section 4.4 in relation to a case study.

Finally, we have looked into what happens to the power and type one error if say two of the treatments have an interesting effect. Using simulation, we devised two scenarios were in the first one both of the experimental treatments have the interesting effect, Δ, but the combination treatment does not, having an uninteresting effect *δ*_0_, thus suggesting that the treatments interact antagonistically with one another, and a second one where only one of the experimental treatments as well as the combination of treatments exhibit the interesting effect, thus implying no interference between the treatments. In the first case where the effect between the experimental treatments is strongly antagonistic, the reduction in power is apparent. Specifically, we notice that the power in the hypothesis testing decreases rapidly as the allocation ratio *q* in the combination treatment group increases. In the second case where only one of the experimental treatments as well as the combination of treatments exhibit an interesting effect, we see that the estimated power is increased.

### 4.4 Case study

For our case study, we use the clinical trial described in Section 2 where we show that in a balanced factorial design, recruiting 45 people per treatment group ensures power of 95*%*. In this section, we investigate the properties of a factorial, an MA and an MAMS design, for different alternative hypothesis scenarios for this design. Using the protocol information on the clinically relevant treatment effect (Δ = 28) and the effect standard deviation *σ* = 50 of the designed clinical trail on the use of physiotherapy on OA 26, we have also assumed that the threshold of a small uninteresting effect is one quarter of the interesting effect (*δ*_0_ = 7). The power for all hypothetical alternative scenarios is calculated by the trivariate normal with the corresponding mean and variance‐covariance matrix for both the factorial and MA design. The Monte Carlo estimate of the expected value based on 10 000‐fold simulations is used for a two‐stage MA design with an O'Brien–Fleming efficacy boundary and a futility bound of 0. The required boundaries are specified such that the pre‐specified power level 1 − *β* = 0.95 is ensured under the alternative hypothesis, by the computation of the probability to reject the null hypothesis given that the alternative is true, and that the FWER is set to *α* = 0.05.

#### 4.4.1 Direct power comparison between a factorial, a multi‐arm and a multi‐arm multi‐stage design

**Figure 5:**
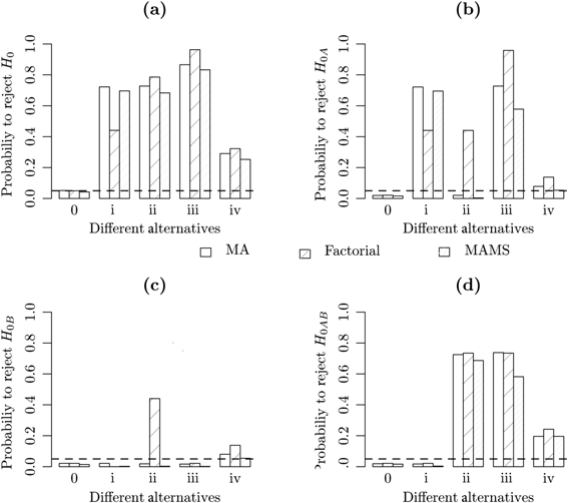
In each of the four plots the probability of rejecting the null hypothesis is depicted for multi‐arm, factorial and multi‐arm multi‐stage designs for all five alternative hypothesis scenarios when (a) the global null hypothesis is rejected (b) the hypothesis for experimental treatment A is rejected (c) the hypothesis for experimental treatment B is rejected (d) the hypothesis for the combination treatment is rejected. We depict with the dashed line the type I error reference level at 0.05.

We investigate the probability of rejecting the null hypotheses for a factorial, an MA and an MAMS design under five different types of alternatives in Figure 5. Scenario (0) *μ*_0_ = *μ*_*A*_ = *μ*_*B*_ = *μ*_*AB*_ = 0 is investigating the global null hypothesis where none of the treatments has an effect. Scenario (i) uses *μ*_*A*_ − *μ*_0_ = Δ,*μ*_*B*_ − *μ*_0_ = *μ*_*AB*_ − *μ*_0_ = *δ*_0_ which is consistent with a LFC setting where one of the single experimental treatments has an interesting effect, whereas the other and the combination treatment have an uninteresting effect. Scenario (ii) investigates another case that is consistent with the LFC (*μ*_*A*_ − *μ*_0_ = *μ*_*B*_ − *μ*_0_ = *δ*_0_,*μ*_*AB*_ − *μ*_0_ = Δ) whilst scenario (iii) *μ*_*A*_ − *μ*_0_ = Δ,*μ*_*B*_ − *μ*_0_ = *δ*_0_,*μ*_*AB*_ − *μ*_0_ = Δ + *δ*_0_ is consistent with the factorial design assumptions. In the final scenario, we are considering (iv) *μ*_*A*_ − *μ*_0_ = *δ*_0_,*μ*_*B*_ − *μ*_0_ = *δ*_0_,*μ*_*AB*_ − *μ*_0_ = 2*δ*_0_ both single experimental treatments have an uninteresting effect, and the strongest effect is in the combination treatment which may still be uninteresting and is consistent with the factorial design assumptions.

Figure 5 demonstrates that under the factorial design the probability of rejecting a hypothesis where the treatment does not have an interesting effect is unreasonably high. We notice that plot (a) of Figure 5 demonstrates how all designs perform the same way under the global null hypothesis (0) and in the case where the treatments have uninteresting effects (iv). At the same time, we can see that the factorial design is highly powered under scenario (iii) which is consistent with the factorial design assumptions, but we can see that it is greatly underpowered in scenario (i), which is a LFC setup. Specifically in this scenario, we observe an antagonistic effect between the experimental treatments and an interaction effect which would imply that *β*_3_ =− Δ in the full linear model setting described in Section 3.1. Finally, we notice that in general, the MA design is more empowered compared with the MAMS design throughout, without however noticing big differences in general. The biggest difference occurs when the factorial assumptions are met, and we assume a strong treatment effect alternative, scenario (iii). One can also observe that under scenario (ii), the factorial design has the largest overall power. This is, however, due to a large chance of rejecting the individual treatment hypothesis despite them not having an interesting effect. Their effect has been increased by borrowing information from the combination arm that truly has an interesting effect in this setting.

**Figure 6:**
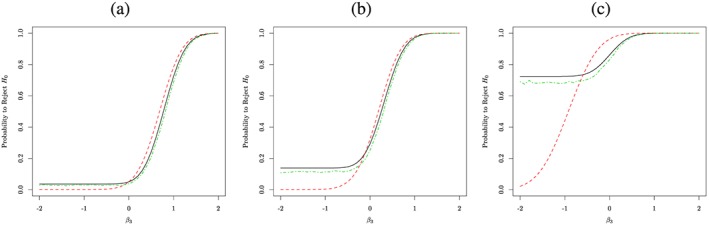
The probability of rejecting the global null hypothesis is depicted for a multi‐arm, a factorial and an multi‐arm multi‐stage design with a black, a red and a green line respectively, for a varying degree of interaction between the treatments in each of the plots, for a different sole treatment level effect pair. We have assumed in (a) μ_A_ − μ_0_ = μ_B_ − μ_0_ = 0, (b) μ_A_ − μ_0_ = μ_B_ − μ_0_ = 7 and (c) μ_A_ − μ_0_ = 0&μ_B_ − μ_0_ = 28.

To investigate the impact of the interaction further, Figure 6 considers different combinations for the mean effect on the single treatment arms ((a) where the sole experimental treatment effects are assumed to be 0, that is *μ*_*A*_ − *μ*_0_ = *μ*_*B*_ − *μ*_0_ = 0, (b) where *μ*_*A*_ − *μ*_0_ = *μ*_*B*_ − *μ*_0_ = 7 and (c) where *μ*_*A*_ − *μ*_0_ = 0&*μ*_*B*_ − *μ*_0_ = 28, whilst the interaction ranges from  − 2 to 2. We find that in the case of no effect in the single treatments, the MA and MAMS design maintain the type I error level for antagonistic effects whilst the factorial design is conservative whereas for a synergistic effect the factorial design has a slightly increased power over both the MA and MAMS design. For all other scenarios, the power of both the MA and MAMS designs is larger (sometimes by a large margin) than for the factorial design when there is an antagonistic effect. For values of *β*_3_ close to zero a slightly larger power for the factorial design is observed, whilst large synergistic effects lead to no difference between the methods. Moreover, we notice a small persistent difference in power between the MA and MAMS design for most values of *β*_3_, especially for strong antagonistic effects, with the exception of large synergistic effects, where there is no difference.

#### 4.4.2 Design differences between an MAMS, a multi‐arm and a factorial trial

Multi‐arm multi‐stage designs will in general be leading to a larger maximum sample size but smaller expected sample size compared with MA ones. The expected sample size is the expected number of patients required to detect a pre‐specified treatment effect and accounts for early stopping at the first stage of the analysis. Because an optimal design has the lowest expected sample size for a given treatment effect, the expected sample size can be used as a measure of the efficiency of the design. In practice, it has been obtained through the evaluation of the expectation of 100 000‐fold simulations of such designs. We do not consider a multi‐stage for a factorial design as it is not possible to stop any treatment early either for efficacy or futility, because all treatments contribute to the computation of the sole treatments' effect. Our previous analysis in Section 4.4.1 revealed a similarity in terms of the relative merits between an MAMS versus factorial and MA versus factorial designs regarding the type I error and power, despite the small deficiency in power of the MAMS design when compared with the MA one. Therefore, the only remaining feature of the MAMS design that is of interest is the expected sample size.

We use the protocol information of our case study trial to see the differences in the total sample size that different choices of clinical trial designs result to, for varying degrees of interaction between the sole experimental treatments. We compare a balanced factorial design, an MA design and an MAMS design with two stages, assuming parameters *σ* = 50, interesting treatment effect Δ = 28, uninteresting treatment effect *δ*_0_ = 7 and *α* = 0.05,1 − *β* = 0.9 for all of them. We have chosen an MA two‐stage design with a 0 futility boundary, an O'Brien–Fleming efficacy boundary 23 and equal sample sizes for all treatments and control for each stage. Thus by implication, the interim analysis is conducted at the half‐way point of the process, and a treatment is dropped if it performs worse than the control, whereas the trial stops early if any treatment's performance exceeds the efficacy boundary, and the null hypothesis is rejected with the conclusion that this treatment is superior to control. The resulting sample size per treatment at the first stage is 38 and cumulatively at the second is 76. The critical value for the upper bound in the first stage is 2.932 and 2.073 for the second. The critical value for the lower bound in the first stage was set to 0 and for the second it is 2.073.

**Figure 7:**
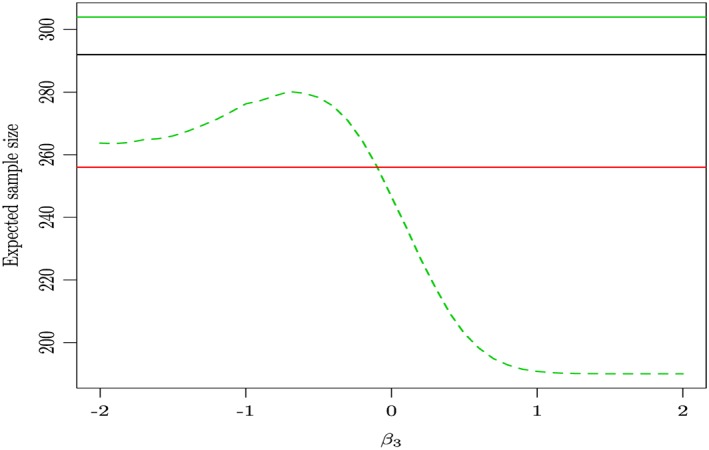
The total trial expected sample size estimate for a two‐stage multi‐arm design (green dotted line) as well as its maximum sample size (solid green line), the sample size of a balanced factorial design (red solid line) and the sample size of a multi‐arm design (solid black line) for varying levels of interaction between the sole experimental treatments when β_3_∈[ − 2,2] for target power 1 − β = 0.9.

Figure 7 interestingly shows that there is no difference in the expected sample size of an MAMS trial and a factorial one when there is no interaction between the treatments. The sample size requirement of an MAMS study increases when the treatments interact antagonistically and decreases when they interact synergistically compared with that of a factorial design. Finally, the maximum sample size of an MAMS study is larger than the sample size required by a simpler MA trial.

## 5 Discussion

The apparent need for efficient clinical trial designs that quickly identify potentially effective treatments has led to the use of designs that simultaneously study many treatments. In this paper, we compared MAMS designs to a factorial with FWER control, and investigated their properties, in terms of their effectiveness in finding good working treatments through their power characteristics under different hypothetical scenarios. We found that the adoption of a factorial design which simply assumes no interaction between the sole treatments may even for modest antagonistic interaction between the sole treatments reduce the power of a hypothesis testing substantially when compared with an MA design. At the same time, we noticed an increase in the type one error rate in the cases of a moderate synergistic interaction between the sole treatments. Furthermore, we have become aware of the role that the choices of allocation ratios to each treatment have on the choice of critical value and the sample size required to achieve a certain target power in the cases both when the factorial assumptions are met or not. The level to which the necessary sample size changes for different allocation ratio combinations is greatly increased in alternative hypothesis scenarios with some degree of interaction between the sole treatments. Finally, through our case study analysis, we discovered that in the scenarios when the factorial assumptions hold, the gain of using this design over an MA in terms of power is small, whereas the losses in power when the assumptions are not satisfied are substantial. In addition, when we include a two‐stage MA design in a comparison of the necessary sample size for a nominal level of target power amongst factorial and MA designs, we find that even in the absence of any interaction the expected trial sample size is the same as that of the factorial, a design that according to previous results in the presence of antagonistic interaction is greatly underpowered.

Based on these results, it is apparent that factorial designs should only really be considered instead of an MA design when the researchers are very sure that the assumption of additivity is met. As soon as there is some doubt, MAMS designs provide a robust alternative that looses little power compared with a factorial design but can gain drastically in other situations. MAMS designs also seem to be preferable to MA designs in such situations as despite their small deficiency in power, they are expected to require a much smaller sample size.

## Appendix A Nested and full linear model design matrix relevant quantities

### A.1

Equation ((A1)) shows the inverse of ***X*** ^⊤^ ***X***, where *d* *e* *t*(***X*** ^⊤^ ***X***) is its determinant and *d* *e* *t*(***X*** ^⊤^ ***X***) = *n*_0_ *n*_*A*_(*n*_*B*_ + *n*_*A**B*_) + *n*_*B*_ *n*_*A**B*_(*n*_0_ + *n*_*A*_) of the nested model in Equation ((3)).

(A1) $${\left(X^{⊤}X\right)}^{-1}=\frac{1}{det(X^{⊤}X)}\left(\begin{array}{lll} n_{A}n_{B}+n_{A}n_{AB}+n_{B}n_{AB} & -n_{A}(n_{B}+n_{AB}) & -n_{B}(n_{A}+n_{AB})\\ -n_{A}(n_{B}+n_{AB}) & \left(n_{0}+n_{A})(n_{B}+n_{AB}\right) & n_{B}n_{A}-n_{0}n_{AB}\\ -n_{B}(n_{A}+n_{AB}) & n_{B}n_{A}-n_{0}n_{AB} & \left(n_{A}+n_{AB})(n_{0}+n_{B}\right) \end{array}\right)$$

Furthermore, using the information on the number of people in each treatment group, we calculate the inverse of ***X*** ^⊤^ ***X*** (Equation ((A2))) of the full model presented first in Section 3.1.

(A2) $${\left(\;X^{⊤}X\;\right)}^{-1}=\left(\begin{array}{llll} \frac{1}{n_{0}} & -\frac{1}{n_{0}} & -\frac{1}{n_{0}} & \frac{1}{n_{0}}\\ -\frac{1}{n_{0}} & \frac{n_{0}+n_{A}}{n_{0}n_{A}} & \frac{1}{n_{0}} & -\frac{n_{0}+n_{A}}{n_{0}n_{A}}\\ -\frac{1}{n_{0}} & \frac{1}{n_{0}} & \frac{n_{0}+n_{B}}{n_{0}n_{B}} & -\frac{n_{0}+n_{B}}{n_{0}n_{B}}\\ \frac{1}{n_{0}} & -\frac{n_{0}+n_{A}}{n_{0}n_{A}} & -\frac{n_{0}+n_{B}}{n_{0}n_{B}} & \frac{n_{B}n_{AB}(n_{A}+n_{AB})+n_{A}n_{AB}(n_{0}+n_{B})}{n_{0}n_{A}n_{B}n_{AB}} \end{array}\right)$$

## Appendix B Derivation of joint distribution of test statistics

### B.1

We show that the joint distribution of ***Z*** = (*Z*_*A*_,*Z*_*B*_,*Z*_*A**B*_) is a trivariate normal by expressing these dependent random variables in terms of linear combinations of the independent variables ${\bar{Y}}_{j}∼N(\mu_{j},\frac{\sigma^{2}}{n_{j}})$ for *j* = *A*,*B*,*A* *B*,0, with *n*_*A*_ = *n*_*B*_ = *r* *n*_0_ and *n*_*A**B*_ = *q* *n*_0_, which precisely due to their independence property, are known to be jointly distributed as a multivariate normal. Specifically, for $\bar{Y}=({\bar{Y}}_{0},{\bar{Y}}_{A},{\bar{Y}}_{B},{\bar{Y}}_{AB})$ we have, $\bar{Y}∼N_{4}(\mu ,\Sigma )$, where ***μ*** = (*μ*_0_,*μ*_*A*_,*μ*_*B*_,*μ*_*A**B*_) and $\Sigma =\left(\begin{array}{llll} \frac{\sigma^{2}}{n_{0}} & 0 & 0 & 0\\ 0 & \frac{\sigma^{2}}{n_{A}} & 0 & 0\\ 0 & 0 & \frac{\sigma^{2}}{n_{B}} & 0\\ 0 & 0 & 0 & \frac{\sigma^{2}}{n_{AB}} \end{array}\right)$. Thus,

$$\begin{array}{cc} Z_{A} & =d_{1}{\bar{Y}}_{A}-d_{2}{\bar{Y}}_{B}+d_{2}{\bar{Y}}_{AB}-d_{1}{\bar{Y}}_{0}\\ Z_{B} & =-d_{2}{\bar{Y}}_{A}+d_{1}{\bar{Y}}_{B}+d_{2}{\bar{Y}}_{AB}-d_{1}{\bar{Y}}_{0}\\ Z_{AB} & =d_{3}{\bar{Y}}_{AB}-d_{3}{\bar{Y}}_{0} \end{array}$$

where $d_{1}=\frac{(r+q)\sqrt{rn_{0}}}{\sigma \sqrt{\left(1+r)(r+q)(r+2q+rq\right)}},d_{2}=\frac{q(1+r)\sqrt{rn_{0}}}{\sigma \sqrt{\left(1+r)(r+q)(r+2q+rq\right)}}$ and $d_{3}=\frac{q\sqrt{n_{0}}}{\sigma \sqrt{1+q}}$. We can express $Z=D\bar{Y}$, using matrix $D=\left(\begin{array}{llll} -d_{1} & d_{1} & -d_{2} & d_{2}\\ -d_{1} & -d_{2} & d_{1} & d_{2}\\ -d_{3} & 0 & 0 & d_{3} \end{array}\right)$ which defines the linear combination of vector $\bar{Y}$. Utilising the basic property of the multivariate normal distribution (MVN) whereby any linear combination of $\bar{Y}$ is also MVN, we see that $Z∼N_{3}(D\mu ,D\Sigma D^{⊤})$. Let ***μ***_*Z*_ = ***D*** ***μ***, therefore for each element of $\mu_{Z}=(\mu_{Z_{A}},\mu_{Z_{B}},\mu_{Z_{AB}})$, we have:

$$\begin{array}{cc} \mu_{Z_{A}} & =\frac{\sqrt{rn_{0}}\left(\right.(r+q)(\mu_{A}-mu_{0})+q(1+r)(\mu_{AB}-\mu_{B})}{\sigma \sqrt{\left(1+r)(r+q)(r+2q+rq\right)}}\\ \mu_{Z_{B}} & =\frac{\sqrt{rn_{0}}\left(\right.(r+q)(\mu_{B}-mu_{0})+q(1+r)(\mu_{AB}-\mu_{A})}{\sigma \sqrt{\left(1+r)(r+q)(r+2q+rq\right)}}\\ \mu_{Z_{AB}} & =\frac{\sqrt{qn_{0}}(\mu_{AB}-\mu_{0})}{\sigma \sqrt{(}1+q)} \end{array}$$

Equation ((B1)) shows the general form of a variance–covariance matrix ***D*** **Σ** ***D*** ^⊤^ = *V*. We find that Var(*Z*_*A*_) = Var(*Z*_*B*_) = Var(*Z*_*A**B*_) = 1 and Cov(*Z*_*A*_,*Z*_*A**B*_) = Cov(*Z*_*B*_,*Z*_*A**B*_), and in the following equations, we present some of the work in deriving Cov(*Z*_*A*_,*Z*_*B*_),Cov(*Z*_*A*_,*Z*_*A**B*_) under the null hypothesis where Cov(*Z*_*A*_,*Z*_*B*_) = *E*(*Z*_*A*_ *Z*_*B*_) − *E*(*Z*_*A*_)*E*(*Z*_*B*_) = *E*(*Z*_*A*_ *Z*_*B*_) and Cov(*Z*_*A*_,*Z*_*A**B*_) = *E*(*Z*_*A*_ *Z*_*B*_) − *E*(*Z*_*A*_)*E*(*Z*_*B*_) = *E*(*Z*_*A*_ *Z*_*B*_).

(B1) $$V=\left(\begin{array}{lll} \mathrm{Var}(Z_{A}) & \text{Cov}(Z_{A},Z_{B}) & \text{Cov}(Z_{A},Z_{AB})\\ \text{Cov}(Z_{B},Z_{A}) & \mathrm{Var}(Z_{B}) & \text{Cov}(Z_{B},Z_{AB})\\ \text{Cov}(Z_{AB},Z_{A}) & \text{Cov}(Z_{B},Z_{AB}) & \mathrm{Var}(Z_{AB}) \end{array}\right)$$

Equations ((B2)) and ((B3)) explicitly show the computations. We also find, using that *V* = ***D*** **Σ** ***D*** ^⊤^, the formulae remain the same under any alternative hypothesis scenario.

(B2) $$\begin{array}{cc} \text{Cov}(Z_{A},Z_{B}) & \;=\;E\left(\right.\frac{rn_{0}[(r+q)({\bar{Y}}_{A}-{\bar{Y}}_{0})+q(1+r)({\bar{Y}}_{AB}-{\bar{Y}}_{B})][(r+q)({\bar{Y}}_{B}-{\bar{Y}}_{0})+q(1+r)({\bar{Y}}_{AB}-{\bar{Y}}_{A}]}{\sigma^{2}(1+r)(r+q)(r^{2}+2rq+r^{2}q)})\\ \;=\;n_{0}\left(\right.\frac{r{\left(r+q\right)}^{2}E[({\bar{Y}}_{A}-{\bar{Y}}_{0})({\bar{Y}}_{B}-{\bar{Y}}_{0})]}{\sigma^{2}(1+r)(r+q)(r+2q+rq)}+\frac{q^{2}r{\left(1+r\right)}^{2}E[({\bar{Y}}_{AB}-{\bar{Y}}_{B})({\bar{Y}}_{AB}-{\bar{Y}}_{A})]}{\sigma^{2}(1+r)(r+q)(r+2q+rq)}\\ \;\;+\frac{qr(r+q)(1+r)\{E[({\bar{Y}}_{A}-{\bar{Y}}_{0})({\bar{Y}}_{AB}-{\bar{Y}}_{A})]+E[({\bar{Y}}_{AB}-{\bar{Y}}_{B})({\bar{Y}}_{B}-{\bar{Y}}_{0})]\}}{\sigma^{2}(1+r)(r+q)(r+2q+rq)})\\ \;=\;\frac{n_{0}\left[\right.r{\left(r\;+\;q\right)}^{2}\left(\right.E(\bar{Y_{0}^{2}})\;-\;\mu^{2})\;+\;q^{2}r{\left(1\;\;+\;r\right)}^{2}\left(\right.E({\bar{Y}}_{AB}^{2})\;-\;\mu^{2})\;+\;qr(r\;+\;q)(1\;+\;r)\left(\right.2\mu^{2}\;-\;E(\bar{Y_{A}^{2}})\;-\;E(\bar{Y_{B}^{2}}))]}{\sigma^{2}(1+r)(r+q)(r+2q+rq)}\\ \;=\;\frac{r{\left(r+q\right)}^{2}+qr{\left(1+r\right)}^{2}-2q(1+r)(r+q)}{\left(1+r)(r+q)(r+2q+rq\right)} \end{array}$$

(B3) $$\begin{array}{cc} \text{Cov}(Z_{A},Z_{AB}) & =\frac{\sqrt{q}n_{0}E\left[\right.\left(\right.r(r+q)({\bar{Y}}_{A}-{\bar{Y}}_{0})+qr(1+r)({\bar{Y}}_{AB}-{\bar{Y}}_{B}))\left(\right.{\bar{Y}}_{AB}-{\bar{Y}}_{0})]}{\sigma^{2}\sqrt{\left(1+r)(1+q)(r+q)(r^{2}+2rq+r^{2}q\right)}}\\ =\frac{\sqrt{q}n_{0}\left(\right.E[r(r+q)({\bar{Y}}_{A}-{\bar{Y}}_{0})({\bar{Y}}_{AB}-{\bar{Y}}_{0})]+E[qr(1+r)({\bar{Y}}_{AB}-{\bar{Y}}_{B})({\bar{Y}}_{AB}-{\bar{Y}}_{0})])}{\sigma^{2}\sqrt{\left(1+r)(1+q)(r+q)(r^{2}+2rq+r^{2}q\right)}}\\ =\frac{\sqrt{q}n_{0}\left(\right.r(r+q)\mathrm{Var}({\bar{Y}}_{0})+qr(1+r)\mathrm{Var}({\bar{Y}}_{AB}))}{\sigma^{2}\sqrt{\left(1+r)(1+q)(r+q)(r^{2}+2rq+r^{2}q\right)}}\\ =\frac{\sqrt{q}n_{0}\left(\right.r(r+q)\frac{\sigma^{2}}{n_{0}}+qr(1+r)\frac{\sigma^{2}}{qn_{0}})}{\sigma^{2}\sqrt{\left(1+r)(1+q)(r+q)(r^{2}+2rq+r^{2}q\right)}}\\ =\frac{\sqrt{rq}(1+2r+q)}{\sqrt{\left(1+r)(1+q)(r+q)(r+2q+rq\right)}} \end{array}$$

Equation ((B4)) shows the variance–covariance matrix, a balanced factorial design.

(B4) $$V=\left(\begin{array}{lll} 1 & 0 & \frac{1}{\sqrt{2}}\\ 0 & 1 & \frac{1}{\sqrt{2}}\\ \frac{1}{\sqrt{2}} & \frac{1}{\sqrt{2}} & 1 \end{array}\right)$$
